# Risk factors of pancreatitis after endoscopic retrograde cholangiopancreatography in patients with biliary tract diseases

**DOI:** 10.1186/s12893-023-01953-4

**Published:** 2023-03-23

**Authors:** Jin-yuan Chi, Lin-ya Ma, Jia-cheng Zou, Yue-feng Ma

**Affiliations:** Department of Biliary Minimally Invasive Surgery, Affiliated Zhongshan Hospital of Dalian University, 116001 Dalian, Liaoning, P. R. China

**Keywords:** Biliary tract disease, Endoscopic retrograde cholangiopancreatography, Risk factors, Postoperative pancreatitis

## Abstract

**Background:**

To investigate the risk factors of pancreatitis after endoscopic retrograde cholangiopancreatography (ERCP) in patients with biliary tract diseases.

**Methods:**

We retrospectively analyzed the clinical data of 480 patients who underwent ERCP for biliary tract diseases at the Affiliated Zhongshan Hospital of Dalian University from October 2011 to October 2016. The patients were divided into a study group (n = 75, with PEP) and a control group (n = 405, without PEP) based on whether they developed post-ERCP pancreatitis (PEP), and their clinical baseline data and intraoperative conditions were retrieved and compared. Then, factors associated with PEP were analyzed using logistic regression model, based on which a nomogram prediction model was constructed. The receiver operating characteristic (ROC) curve and calibration curve were used to evaluate the performance of the prediction model.

**Results:**

Significant differences in age, sex, history of pancreatitis, history of choledocholithiasis, pancreatic duct imaging, pancreatic sphincterotomy, difficult cannulation, multiple cannulation attempts and juxtapapillary duodenal diverticula were observed between the two groups. Multivariate logistic regression analysis showed that age less than 60 years (OR, 0.477; 95% CI, 0.26–0.855), female sex (OR, 2.162; 95% CI, 1.220–3.831), history of pancreatitis (OR, 2.567; 95% CI, 1.218–5.410), history of choledocholithiasis (OR, 2.062; 95% CI, 1.162–3.658), pancreatic sphincterotomy (OR, 2.387; 95% CI, 1.298–4.390), pancreatic duct imaging (OR, 4.429; 95% CI, 1.481–13.242), multiple cannulation attempts (OR, 2.327; 95% CI, 1.205–4.493), difficult cannulation (OR, 2.421; 95% CI, 1.143–5.128), and JPD (OR, 2.002; 95% CI, 1.125–3.564) were independent risk factors for PEP. The nomogram for predicting the occurrence of PEP demonstrated an area under the ROC curve (AUC) of 0.787, and the calibration curves of the model showed good consistency between the predicted and actual probability of PEP.

**Conclusion:**

Our results showed that age less than 60 years, female sex, history of pancreatitis, history of choledocholithiasis, pancreatic sphincterotomy, pancreatic duct imaging, multiple cannulation attempts, difficult cannulation and juxtapapillary duodenal diverticula were independent risk factors for PEP. In addition, the established nomogram demonstrated promising clinical efficacy in predicting PEP risk in patients who underwent ERCP for biliary tract diseases.

## Background

Endoscopic retrograde cholangiopancreatography (ERCP) was first performed by McCune et al. in 1968 [1], and the first successful case in China was reported in 1973 [2]. This procedure is now commonly used to diagnose and treat biliary tract and pancreatic diseases [3]. Although ERCP is considered safe and effective in treating biliary tract diseases [4, 5], its rate of complications and mortality of ERCP over a decade was reported to be 10-12% and 0.4-1.4%, respectively [6–8].

The main complications after ERCP are pancreatitis, bleeding, cholecystitis, infection and intestinal perforation. Among them, post-ERCP pancreatitis (PEP) is the most common, with an incidence between 1.6 and 15% [9, 10]. Most cases of PEP are mild or moderate, and only a few present with severe conditions. Epidemiological studies showed that about 1.5% of PEP cases were moderate to severe, and the overall mortality rate of PEP was approximately 3% [9]. In severe cases, PEP can lead to multiple organ dysfunction, thereby increasing the risk of treatment-related mortality. The treatment of PEP requires substantial healthcare resources and usually needs prolonged hospitalization, increasing the psychosocial burden of endoscopists [11, 12]. Thus, timely identifying related risk factors could be critical for preventing and reducing the incidence of PEP.

In this study, we used univariate and multivariate analysis to identify the influence of patient baseline data and intraoperative conditions on the incidence of PEP and screened out independent risk factors to build a predicting score model that clinicians could use for targeted treatment and interventions.

## Materials and methods

### General information

This study was approved by the Ethics Committee of the Affiliated Zhongshan Hospital of Dalian University, following the clinical data of patients who underwent ERCP for biliary tract diseases from October 2011 to October 2016 were retrospectively analyzed. The study inclusion criteria were [13]: (1) aged 18–80 years old; (2) the diagnosis of PEP was made following the criteria developed by Cotton et al. in 1991 [14]: occurrence of persistent pancreatitis-related symptoms (i.e., new or aggravated abdominal pain) after ERCP, accompanied by an increase in serum amylase level by more than 3 times the normal limit within 24 h after surgery, and requiring hospitalization for more than 1 day; (3) patients with normal serum amylase or < 3 times higher than the normal limit before ERCP; and (4) patients with complete preoperative biochemical examinations for blood and urine, liver and kidney function tests, hematuria amylase, blood lipids and blood glucose, as well as electrocardiogram, abdominal color ultrasound or computed tomography, and postoperative examinations of blood biochemistry, serum amylase, liver function and others. The exclusion criteria were: (1) patients with benign and malignant tumors of the biliopancreatic system; (2) the presence of benign and malignant duodenal stenosis; (3) non-native papilla (including post-sphincterotomy, post-papillectomy, post-papillary balloon dilatation, and post-choledochojejunostomy); and (4) missing indicators that could have affected the main statistical analysis of this study.

### ERCP procedure

All ERCP procedures in this study were performed by experienced teams, which included an endoscopist with at least 100 successfully completed ERCP per year and 10 or more years of experience, an endoscopy nurse adequately skilled in the technique, and a physician. The patients were orally anesthetized using a 10 mL dyclonine capsule for throat mucosa anesthesia and intravenous combined anesthesia with 3–5 mg/kg per hours propofol pump for those intolerant. Fujitcan’s duodenoscope system ED-530 XT electronic endoscopic system was used, tentative insertion along the oesophagus, enter the duodenal bulb, incorporating the duodenal papilla into the center of the field of view, observed the morphology and structure of the nipple in detail. The selection of bile and pancreatic duct cannulation was according to the size and morphology of the duodenal papilla, opening and wrinkle, local presence or absence of diverticula and the relationship between the duodenal papilla and diverticulum. In cases of difficult intubation, the precut sphincterotomy or needle knife was employed to gain access to the common bile duct (CBD) when standard methods using catheters, cannulatomes and guidewires have failed. After successful intubation, bile and pancreatic fluid was withdrawn and then appropriate contrast agent was injected. According to the patient’s condition, endoscopic sphincterotomy (EST), duodenal papillary balloon dilatation, net basket or balloon stone extraction, pancreatic duct stent placement or bile duct stent placement was performed. All included patients were treated with prophylactic 100 mg rectal indomethacin before the procedure.

### Baseline indicators

Baseline data were collected from all patients, including age, sex, history of pancreatitis, history of diabetes, history of hypertension, history of previous ERCP, history of previous cholecystectomy, history of choledocholithiasis, total bilirubin, and triglyceride levels. Whether patients had the following intraoperative conditions were observed and recorded, including common bile duct dilatation, pancreatic duct imaging (pancreatic duct can be observed below X ray after contrast injection), EST {the sphincterotomy is extended to expose the biliary lumen and the biliary duct can be cannulated, including pancreatic sphincterotomy, biliary sphincterotomy, duodenal minor papilla sphincterotomy (use a needle-knife or wire-assisted access sphincterotomy to dorsal pancreatic duct drainage when patients with recurrent acute or chronic pancreatitis who have complete or incomplete pancreas divisum)}, duodenal papillary balloon dilatation (a 8-mm diameter balloon was placed in the papilla to dilate, used as an alternative to EST for extracting CBD stones < 8 mm in patients, choledocholithiasis is the only indication), difficult cannulation (According to the European Digestive Endoscopy Association (ESGE) guidelines [15], defined as more than five contacts with the papilla prior to successful cannulation; more than 5 min of cannulation time following visualization of the papilla; and more than one inadvertent pancreatic duct (PD) cannulation or opacification), multiple cannulation attempts (≥ 3 times), operation time more than 60 min, and juxtapapillary duodenal diverticula.

### Statistical analysis

Data analysis was performed using the SPSS software (version 26.0). Count data are presented as n (%), and measurement data as mean ± SD. Comparisons between groups were performed using the χ^2^ test or Fisher’s exact test. Univariate and multivariate logistic regression models were used to analyze the relationship between clinical data and PEP to determine the independent risk factors for PEP. The obtained independent risk factors were used to construct a nomogram model using the “rms” package of the R software, which could be used to assess the risk of PEP. Additionally, a calibration curve and receiver operating characteristic (ROC) curve were drawn to evaluate the performance of the nomogram. *P* < 0.05 was used to indicate a significant difference.

## Results

### Comparison of baseline characteristics between the groups

Based on the inclusion and exclusion criteria, a total of 480 patients were found eligible for this study. Those who developed PEP were assigned to the study group (n = 75), while those who did not develop PEP were assigned to the control group (n = 405). The clinical baseline data of all patients are shown in Table 1. There were significant differences between the study group and control group in age (< 60 years; 45.3% vs. 31.6%), sex (female; 60.0% vs. 37.3%), history of pancreatitis (yes; 26.7% vs. 7.9%), and history of choledocholithiasis (yes; 42.7% vs. 28.9%) (all *P* < 0.05). However, no marked differences were found between the two groups in terms of history of diabetes, hypertension, ERCP and its indications, cholecystectomy, total bilirubin and triglyceride (*P* > 0.05).

**Table 1:** Comparison of baseline data between the two groups

Baseline data	Control group (n = 405)	Study group (n = 75)	X^2^	*P*
Age (%)			5.334	0.021
< 60	128(31.6)	34(45.3)		
≥ 60	277(68.4)	41(54.7)		
Sex (%)			13.516	<0.001
Male	254(62.7)	30(40.0)		
Female	151(37.3)	45(60.0)		
History of pancreatitis (%)			23.069	<0.001
No	373(92.1)	55(73.3)		
Yes	32(7.9)	20(26.7)		
History of diabetes (%)			0.889	0.346
No	321(79.3)	63(84.0)		
Yes	84(20.7)	12(16.0)		
History of hypertension (%)			0.026	0.872
No	277(68.4)	52(69.3)		
Yes	128(31.6)	23(30.7)		
History of ERCP (%)			1.504	0.220
No	285(70.4)	58(77.3)		
Yes	120(29.6)	17(22.7)		
Indication for ERCP (%)				
Choledocholithiasis	203(50.1)	46(61.3)	3.185	0.074
biliary stricture	0(0)	1(1.3)	-	0.156
pancreatitis	39(9.6)	12(16.0)	2.704	0.100
cholangeitis	216(53.3)	45(60.0)	1.134	0.287
Others	110(27.2)	17(22.7)	0.657	0.418
History of cholecystectomy (%)			0.558	0.455
No	358(88.4)	64(85.3)		
Yes	47(11.6)	11(14.7)		
History of choledocholithiasis (%)			5.612	0.018
No	288(71.1)	43(57.3)		
Yes	117(28.9)	32(42.7)		
Total bilirubin	78.93 ± 95.77	65.85 ± 92.76	1.091	0.276
Triglyceride	1.52 ± 0.85	1.45 ± 0.68	0.751	0.453

Count data are expressed as n (%), and measurement data as mean ± SD. ERCP, Endoscopic retrograde cholangiopancreatography; study group, patients developed pancreatitis after ERCP; control group, patients did not develop pancreatitis after ERCP.

### Comparison of intraoperative conditions between the two groups

The intraoperative conditions of patients are shown in Table 2. Compared with the control group, the study group had a significantly higher proportion in pancreatic duct imaging, pancreatic sphincterotomy, pancreatic stents, difficult cannulation, multiple cannulation attempts, and juxtapapillary duodenal diverticula (all *P* < 0.05). However, there was no significant difference between the two groups in intraoperative common bile duct dilatation, biliary sphincterotomy, duodenal papillary sphincterotomy, duodenal papillary balloon dilatation, and operation time exceeding 60 min (all *P* > 0.05).

**Table 2:** Comparison of intraoperative conditions of patients between the two groups

Intraoperative conditions	Control group (n = 405)	Study group (n = 75)	X^2^	*P*
Common bile duct dilatation (%)			0.382	0.537
No	356(87.9)	64(85.3)		
Yes	49(12.1)	11(14.7)		
Pancreatic duct imaging (%)			16.083	<0.001
No	395(97.5)	65(86.7)		
Yes	10(2.5)	10(13.3)		
Pancreatic sphincterotomy (%)			19.079	<0.001
No	322(79.5)	42(56.0)		
Yes	83(20.5)	33(44.0)		
Biliary sphincterotomy (%)			1.757	0.185
No	290(71.6)	48(64.0)		
Yes	115(28.4)	27(36.0)		
Duodenal minor papilla sphincterotomy (%)			1.023	0.312
No	109(26.9)	16(21.3)		
Yes	296(73.1)	59(78.7)		
Duodenal papillary balloon dilatation (%)			0.745	0.388
No	243(60.0)	41(54.7)		
Yes	162(40.0)	34(45.3)		
Pancreatic stents (%)			7.255	0.007
No	335(82.7)	52(69.3)		
Yes	70(17.3)	23(30.7)		
Difficult cannulation (%)			14.294	<0.001
No	372(91.9)	58(77.3)		
Yes	33(8.1)	17(22.7)		
Multiple cannulation attempts (%)			10.268	0.001
< 3 times	347(85.7)	53(70.7)		
≥ 3 times	58(14.3)	22(29.3)		
Operation time (%)			1.583	0.208
<60 min	347(85.7)	60(80.0)		
≥ 60 min	58(14.3)	15(20.0)		
Juxtapapillary duodenal diverticula (%)			15.477	<0.001
No	279(68.9)	34(45.3)		
Yes	126(31.1)	41(54.7)		

Count data are expressed as n (%)

### Risk factors for post-ERCP pancreatitis

Univariate analysis of the general data and intraoperative conditions of the included patients showed that age less than 60, female sex, history of pancreatitis, history of choledocholithiasis, pancreatic sphincterotomy, pancreatic duct imaging, multiple cannulation attempts, difficult cannulation and juxtapapillary duodenal diverticula were significantly associated with PEP (*P* < 0.05), while no significant difference was observed for history of diabetes, hypertension, ERCP and cholecystectomy, total bilirubin, triglyceride, common bile duct dilatation, biliary sphincterotomy, duodenal minor papilla sphincterotomy, duodenal papillary balloon dilatation, pancreatic stents and operation time more than 60 min (*P* > 0.05) (Table 3).

**Table 3:** Univariate analysis of risk factors for post-ERCP pancreatitis

Variables	B	S.E.	Wald	P	OR	95%CI	
						Lower limit	Upper limit
Age (< 60)	-0.585	0.255	5.243	0.022	0.557	0.338	0.919
Sex (female)	0.926	0.257	12.956	<0.001	2.523	1.524	4.177
History of pancreatitis	1.444	0.320	20.427	<0.001	4.239	2.266	7.929
History of diabetes	-0.318	0.338	0.883	0.347	0.728	0.375	1.412
History of hypertension	-0.044	0.272	0.026	0.872	0.957	0.561	1.632
History of ERCP	-0.362	0.296	1.493	0.222	0.696	0.389	1.245
History of cholecystectomy	0.269	0.361	0.556	0.456	1.309	0.645	2.658
History of choledocholithiasis	0.605	0.258	5.508	0.019	1.832	1.105	3.037
Total bilirubin	-0.002	0.001	1.181	0.277	0.998	0.995	1.001
Triglyceride	-0.126	0.167	0.565	0.452	0.882	0.635	1.224
Common bile duct dilatation	0.222	0.360	0.380	0.537	1.249	0.616	2.530
Pancreatic duct imaging	1.804	0.467	14.943	<0.001	6.077	2.434	15.172
Pancreatic sphincterotomy	1.115	0.263	17.934	<0.001	3.048	1.820	5.106
Biliary sphincterotomy	0.350	0.265	1.745	0.186	1.418	0.844	2.383
Duodenal minor papilla sphincterotomy	0.306	0.303	1.017	0.313	1.358	0.749	2.461
Duodenal papillary balloon dilatation	0.218	0.253	0.743	0.389	1.244	0.757	2.043
Pancreatic stents	0.750	0.283	7.031	0.008	2.117	1.216	3.685
Difficult cannulation	1.195	0.330	13.098	<0.001	3.304	1.730	6.312
Multiple cannulation attempts (≥ 3 times)	0.910	0.291	9.798	0.002	2.483	1.405	4.389
Operation time (≥ 60 min)	0.403	0.322	1.567	0.211	1.496	0.796	2.809
Juxtapapillary duodenal diverticulum	0.982	0.256	14.767	<0.001	2.670	1.618	4.406

Multivariate logistic regression analysis (Table 4) was then performed to identify independent factors associated with the risk of developing PEP. The results showed that age less than 60 years (OR, 0.477; 95% CI, 0.26–0.855), female sex (OR, 2.162; 95% CI, 1.220–3.831), history of pancreatitis (OR, 2.567; 95% CI, 1.218–5.410), history of choledocholithiasis (OR, 2.062; 95% CI, 1.162–3.658), pancreatic sphincterotomy (OR, 2.387; 95% CI, 1.298–4.390), pancreatic duct imaging (OR, 4.429; 95% CI, 1.481–13.242), multiple cannulation attempts (OR, 2.327; 95% CI, 1.205–4.493), difficult cannulation (OR, 2.421; 95% CI, 1.143–5.128) and juxtapapillary duodenal diverticula (OR, 2.002; 95% CI, 1.481–13.242) 1.125–3.564) were independent risk factors for PEP (*P* < 0.05).

**Table 4:** Multivariate analysis of risk factors for post-ERCP pancreatitis

Variables	B	S.E.	Wald	P	OR	95%CI	
						Lower limit	Upper limit
Age (< 60)	-0.740	0.298	6.180	0.013	0.477	0.266	0.855
Sex (female)	0.771	0.292	6.983	0.008	2.162	1.220	3.831
History of pancreatitis	0.943	0.380	6.146	0.013	2.567	1.218	5.410
History of choledocholithiasis	0.724	0.293	6.115	0.013	2.062	1.162	3.658
Pancreatic duct imaging	1.488	0.559	7.090	0.008	4.429	1.481	13.242
Pancreatic sphincterotomy	0.870	0.311	7.831	0.005	2.387	1.298	4.390
Difficult cannulation	0.884	0.383	5.330	0.021	2.421	1.143	5.128
Multiple cannulation attempts (≥ 3 times)	0.845	0.336	6.328	0.012	2.327	1.205	4.493
Juxtapapillary duodenal diverticulum	0.694	0.294	5.570	0.018	2.002	1.125	3.564

### Establishment and validation of a predicting model for estimating PEP risk

**Fig. 1:**
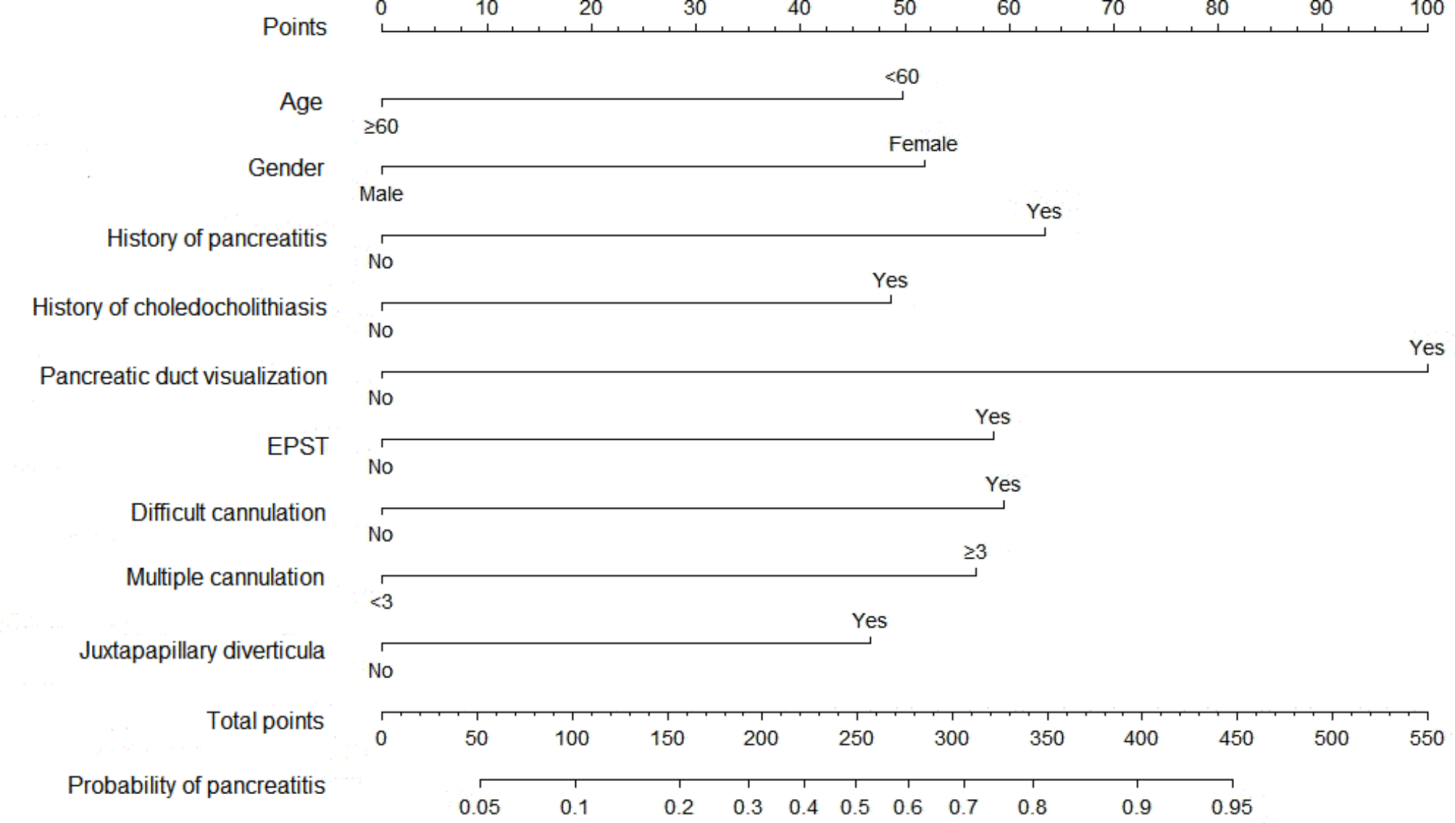
Nomogram for predicting the probability of post-ERCP pancreatitis Abbreviations: EPST, endoscopic pancreatic sphincterotomy.

**Fig. 2:**
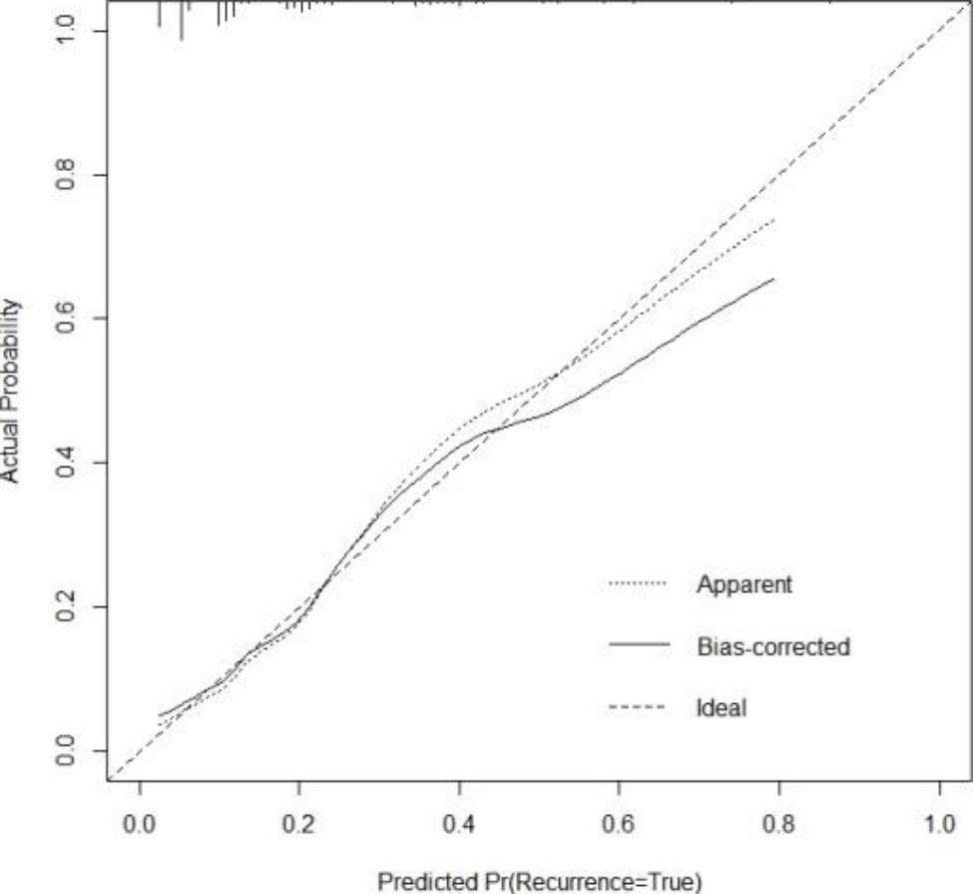
Calibration plot of the constructed nomogram Note: Apparent indicates the prediction curve without calibration; Bias-corrected indicates the calibrated prediction curve; Ideal represents the standard curve; predicted Pr is the predicted incidence of post-ERCP pancreatitis; and Actual Probability indicates the actual incidence of post-ERCP pancreatitis

A nomogram comprising nine covariates was constructed based on the results of the multivariate logistic regression model (Fig. 1). Each risk factor was assigned a score, and the probability of PEP was inferred by summing the scores of all factors. As shown in Fig. 2, the standard curve fitted well with the calibrated prediction curve, indicating satisfactory consistency between the predicted and the actual incidence of PEP in our study cohort.

### Receiver operator characteristic (ROC) curve

**Fig. 3:**
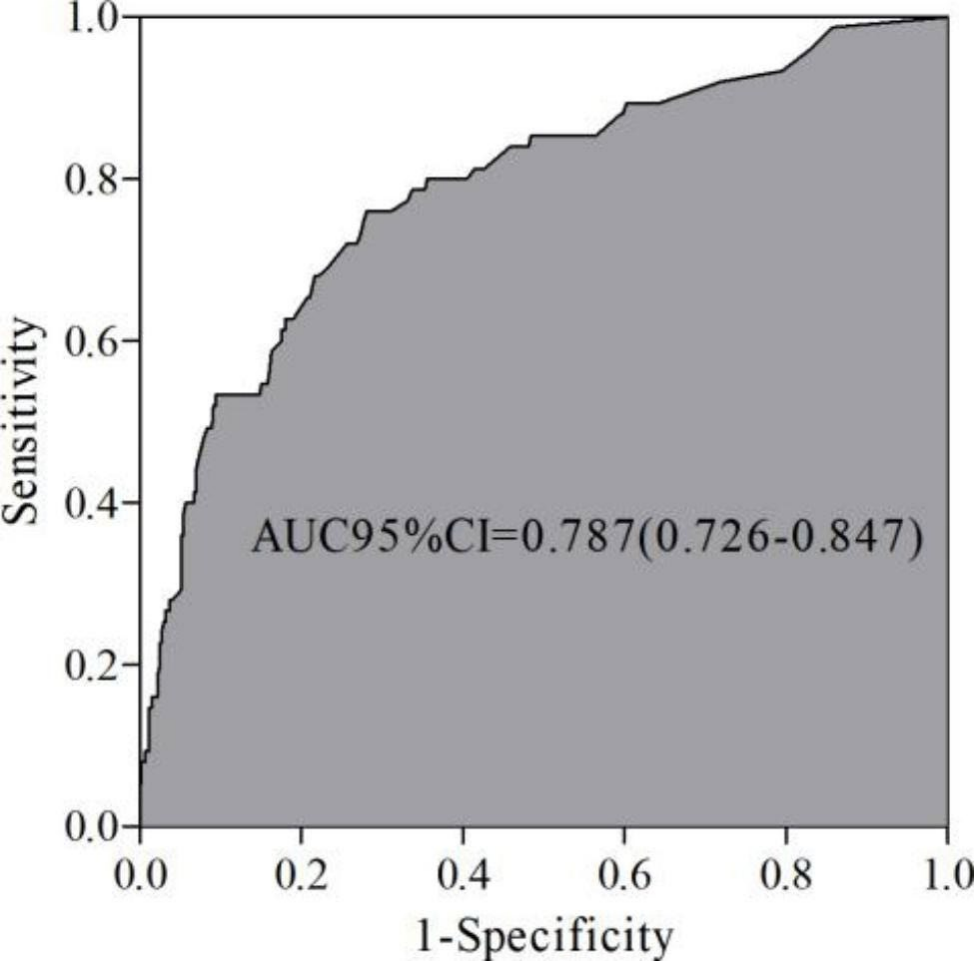
Receiver operator characteristic (ROC) curve

The performance of the nomogram model in predicting the risk of PEP was assessed by plotting the ROC curve, which showed an AUC of 0.787 and a 95% CI ranging between 0.726 and 0.847, indicating a high predictive value of the prediction model (*P* < 0.05) (Fig. 3).

## Discussion

PEP, a common complication of ERCP, can significantly increase the risk of mortality of the patients and healthcare-associated costs [16], indicating the need to strategize newer strategies to reduce the incidence of post-ERCP complications. However, before developing specific interventions, it is important to understand the risk factors for PEP. More and more influencing factors have been found with the deepening of research, including individual differences of patients, the experience level of endoscopists, and many clinical factors [17]. In this present study, we analyzed the risk factors associated with PEP, based on which we constructed a scoring model that could be used as a reference for the targeted prevention of PEP, thereby reducing its incidence and additional medical costs and improving therapeutic outcomes.

The results showed that there were significant differences between the study group and control group in age, sex, history of pancreatitis, history of choledocholithiasis, pancreatic duct imaging, pancreatic sphincterotomy, pancreatic stents, difficult cannulation, multiple cannulation attempts, and juxtapapillary duodenal diverticula. Further univariate and multivariate analysis confirmed that age (< 60 years), sex (female), history of pancreatitis, history of choledocholithiasis, pancreatic sphincterotomy, pancreatic duct imaging, multiple cannulation attempts, difficult cannulation and juxtapapillary duodenal diverticula were associated with PEP and were independent risk factors for PEP, which was consistent with previous findings [17, 18]. Studies have shown that the prevalence of PEP is significantly higher in patients younger than 60 years of age than in elderly patients [19, 20], possibly due to the decrease in the exocrine function of the pancreas with increasing age. When the pancreas is mildly injured, the body is stressed, leading to a sudden increase in pancreatic juice and further aggravating pancreatic injury. Gender differences can cause different body structures, functions, and hormone secretion levels, resulting in a higher probability of PEP in women than in men [21]. In addition, studies have reported that patients with a history of pancreatitis, choledocholithiasis, intraoperative pancreatic sphincterotomy, and intraoperative pancreatic duct imaging are independent risk factors for PEP [22]. This is because such patients might have an underlying impaired pancreatic juice excretion, making them more likely to suffer from acute pancreatitis after ERCP-caused irritation to the pancreas [23].

Multiple imagings of the pancreatic duct were found to increase the incidence of PEP [24]. Pancreatography increases pressure in the pancreatic duct, resulting in pancreatic juice reflux and increasing the risk of acute pancreatitis post-ERCP. Electrocoagulation performed during pancreatic sphincterotomy can further damage the pancreatic duct, easily leading to acute pancreatitis after ERCP [25]. Therefore, appropriate clinical treatment and intervention can be given to patients with a history of pancreatitis, choledocholithiasis, pancreatic duct imaging, and pancreatic sphincterotomy to reduce the risk of PEP. Difficult cannulation and multiple cannulation attempts are also risk factors for PEP. Repeated attempts at cannulating the duodenal papilla can lead to papillary injury, papillary swelling, sphincter relaxation and contraction dysfunction, hindering the discharge of pancreatic juice from the pancreatic duct and resulting in the accumulation of juice in the pancreatic duct. As a result, the pressure in the pancreatic duct is increased, causing damage to pancreatic duct epithelial cells and acini, activation of pancreatic enzymes, and induction of PEP [26]. Further, it is known that the juxtapapillary duodenal diverticula is a contributor to PEP. Residual food in the duodenal diverticulum or huge diverticulum compresses the biliary and pancreatic sphincters, inducing contraction and cramps of the biliary sphincter and poor bile excretion. Bile and bacteria may remain in the diverticulum, leading to duodenal papillitis, triggering dyskinesia of the sphincter, and then inducing pancreatitis [27].Timely risk assessment, optimally at the onset of acute pancreatitis, is crucial to maximize treatment efficacy, provide optimal management and identify those at high risk of developing complications such as infected necrosis or organ failure, which might ultimately lead to unfavorable treatment and survival outcomes [28–31]. Previously, multiple scoring systems, such as the Harmless acute pancreatitis Score (HAPS) [32], Ranson criteria [33], Balthazar score [34], bedside index for severity of acute pancreatitis (BISAP) [35], PANC3 score [36] and others, were proposed for the early phase of acute pancreatitis to predict the disease course and further outcomes. However, their accuracy tended to vary based on the population selected, institutions performed, etc. [37, 38]. Additionally, there is a lack of scoring model systems, a user-friendly scoring system using clinically readily available data, for estimating the risk of PEP. In this regard, a recent study by Fu et al. were the first to build and validate a risk prediction model, especially for PEP after biliary stent placement due to malignant biliary obstruction (MBO), which demonstrated an AUC of 0.810 and 0.781 in their developmental and validation cohort [39]. However, their study focused only on MBO cases, thereby affecting the generalizability of their results for other cases undergoing ERCP. Zheng et al. recently proposed a scoring system for predicting the risk of PEP, but their study was limited by a lack of cases, external validation, and a limited number of variables in their scoring model [40]. Thus, considering the lack of scoring systems and limitations in the current literature, we undertook this present study to determine the potential use of a scoring model to assist clinicians in identifying high-risk patients and help implement preventive measures more promptly. Our proposed prediction model for PEP demonstrated good agreement with the actual observed values after calibration. The ROC curve (AUC = 0.787) also indicated the promising predictive value of our proposed nomogram.

Despite the interesting findings described, our study had several limitations. First, this was a single-center retrospective study with a relatively small sample size, limiting the generalizability of our proposed nomogram, thus, urging the need for further verification in large-scale, multicenter prospective studies. Second, due to the limited data available, we could not validate the nomogram and hope that in the future, these findings could be validated in populations outside of China. Third, due to the issues in data retrieval, some data, such as operator experience or papilla type, were not investigated.

## Conclusion

In summary, age less than 60 years, sex being female, history of pancreatitis, history of choledocholithiasis, pancreatic sphincterotomy, pancreatic duct imaging, multiple cannulation attempts, difficult cannulation, and juxtapapillary duodenal diverticula are independent risk factors for PEP. Therefore, clinicians need to evaluate these indicators before ERCP to effectively prevent PEP, which could help reduce the incidence of PEP, patients’ mortality and pain and medical resources. Thus, our proposed model could be used as a reference for estimating the risk of these patients to develop PEP, based on which a personalized treatment approach could be used to improve the treatment and survival outcomes of these sufferers.

## Data Availability

The datasets used and/or analyzed during the current study can be made available from the corresponding author on reasonable request.
